# Application of interpretable machine learning algorithms to predict distant metastasis in osteosarcoma

**DOI:** 10.1002/cam4.5225

**Published:** 2022-09-09

**Authors:** Bing‐li Bai, Zong‐yi Wu, She‐ji Weng, Qing Yang

**Affiliations:** ^1^ Department of Orthopedics Surgery The Second Affiliated Hospital and Yuying Children's Hospital of Wenzhou Medical University Wenzhou China; ^2^ Department of Breast Surgery The Second Affiliated Hospital and Yuying Children's Hospital of Wenzhou Medical University Wenzhou China

**Keywords:** adaptive synthetic technique, distant metastasis, machine learning, osteosarcoma, SEER, Shapley additive explanation

## Abstract

**Background:**

Osteosarcoma is well‐established as the most common bone cancer in children and adolescents. Patients with localized disease have different prognoses and management than those with metastasis at the time of diagnosis. The purpose of this study was to explore potential risk factors for metastatic disease.

**Methods:**

The Surveillance, Epidemiology, and End Results (SEER) Program database was used to identify patients diagnosed with osteosarcoma between 2004 and 2015. We developed prediction models for distant metastasis using six machine learning (ML) techniques, including logistic regression (LR), support vector machine (SVM), Gaussian Naive Bayes (GaussianNB), Extreme Gradient Boosting (XGBoost), random forest (RF), and k‐nearest neighbor algorithm (kNN). The adaptive synthetic (ADASYN) technique was used to deal with imbalanced data. The Shapley Additive Explanation (SHAP) analysis generated visualized explanations for each patient. Finally, the average precision (AP), sensitivity, specificity, accuracy, F1 score, precision‐recall curves, calibration plots, and decision curve analysis (DCA) were conducted to evaluate the models' effectiveness.

**Results:**

The six machine learning algorithms achieved AP of 0.661–0.781 for predicting distant metastasis. The RF model yielded the best performance with an accuracy of 71.8 percent and an AP of 0.781 and was highly dependent on tumor size, primary surgery, and age. SHAP analysis provided model‐independent interpretation, highlighting significant clinical factors associated with the risk of metastasis in osteosarcoma patients.

**Conclusions:**

An accurate machine learning‐based prediction model was established for metastasis in osteosarcoma patients to help clinicians during clinical decision‐making.

## Graphical abstract

We developed a machine learning‐based model to predict the explore potential risk factors for metastatic disease in osteosarcoma patients according to the tumor features and the treatment duration in this study. As a result, the Random Forest model outperformed all other models in training and validation sets. To improve the interpretability of our machine learning model, we reported the SHAP values and a list of highly influential features.

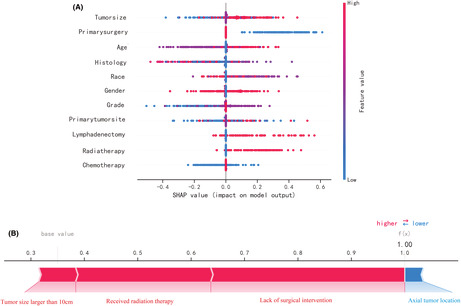

## 1 INTRODUCTION

Osteosarcoma is widely acknowledged as the most common type of primary malignant bone tumor that arises from mesenchymal tissue,^1^ with an incidence rate of 4 to 5 cases per million people.^2^ It has a bimodal age distribution pattern, occurring predominantly in children and young adults <20 years of age and those >50–60 years of age.^3^, ^4^ It has been reported that metastasis disease is found in approximately 20% of osteosarcoma patients at diagnosis.^3^, ^5^ Patients with localized osteosarcoma have a significantly higher 5‐year survival rate than patients with distant metastasis (70% vs. 20%).^6^ Complete surgical resection combined with adjuvant chemotherapy is the best treatment for osteosarcoma patients with distant metastases.^7^ Despite this, the prognosis for metastatic disease remains dismal, with relapse happening in more than half of patients.^8^ Distant metastasis is widely considered as one of the most important long‐term prognostic indicators for osteosarcoma patients. These findings highlight the need to develop technologies that can detect distant metastasis in patients at diagnosis to allow implementation of personalized therapeutic approaches.

Herein, we used the SEER Program database, a commonly used tool for analyzing rare cancers.^9^ The SEER Program is known to collect data from seventeen geographically diverse cancer registries, covering roughly 26% of the United States population. Modern data mining and machine learning approaches can assist in uncovering hidden correlations between parameters by extracting implicit relevant information and expertise from massive amounts of data.^10^ Over the years, machine learning has been successfully used to diagnose and predict many diseases.^11^, ^12^ Accordingly, we gathered a large, international cohort of osteosarcoma patients to develop and validate a machine learning‐based model for analyzing clinical characteristics associated with the risk of distant metastasis.

## 2 METHODS

### 2.1 Study population and variable selection

In the current study, we used data from the SEER database (approval number: 11875‐Nov2020), a population‐based cancer registry at the National Cancer Institute in the United States (SEER*Stat 8.3.6). The inclusion criteria consisted of: (a) patients diagnosed with osteosarcoma as the “primary and only cancer diagnosis” from 2004 to 2015; (b) the ICD‐O‐3 morphology codes “9180–9187,9192–9195”; (c) the primary site of tumors was bone (C400‐C419); (d) patients with evidence of metastasis. The exclusion criteria included patients with (a) multiple primary cancers and (b) missing or blank clinical information. Variables were classified based on their clinical relevance and previously reported thresholds. Patient characteristics of interest included age, gender, race, tumor size, anatomic location, grade, histology, surgery, radiotherapy, chemotherapy, lymphadenectomy, and stage. Patients were separated into different age groups (<24 years old, 24–59 years old, and > 60 years old) and ethnicity (White, Black, and Other ethnic groups). Pathological tumor grade based on the variable “ICD‐O‐3 grade” and classified into grades I, II, III, and IV. The tumor size was stratified as ≤5.0 cm, 5.0–10 cm or >10 cm. The primary tumor location consisted of the axial skeleton, extremities, and others. Our primary outcome variable was a binary variable indicating the presence of metastatic disease at the time of diagnosis. In this category, we included patients with “distant” disease. Patients with “localized” or “regional” staging were considered free of metastasis. The non‐parametric miss‐Forest method was used to impute missing data.^13^ A heatmap was used to visualize Pearson's correlation test results and examine the relationship between variables.

### 2.2 Imbalanced data processing

There were 1891 (22.6%) patients with metastasis and 553 (77.4%) patients without metastasis in the dataset. The large difference between the two classes could lead to low classifier prediction power. Indeed, it is widely acknowledged that balanced data can achieve better prediction performance. ADASYN is a powerful oversampling method widely used in machine learning with imbalanced data.^14^ In our research, we implemented the ADASYN technique using three different percentages 100%, 150%, and 200%. The minority class increased from 22.4 percent in the raw dataset to 46.7 percent in the ADASYN with a 200% dataset as a result of this. (Table 1) Then, this balanced dataset was randomly split into training (70%) and test (30%) sets.

**TABLE 1:** Number of instances increased by ADASYN technique

Percentage of ADASYN increase	Class “No” actual 1819 (76.7%)	Class “Yes” actual 553 (23.3%)
100%	1819	1116
150%	1819	1383
200%	1819	1659

### 2.3 Establishment and evaluation of the predictive model

In this study, six machine learning algorithms were chosen to predict distant metastasis in osteosarcoma patients. The six models that we have developed are as follows: logistic regression model (LR), support vector machine (SVM), Gaussian Naive Bayes (GaussianNB), Extreme Gradient Boosting (XGBoost) model, random forest (RF), and k‐nearest neighbor algorithm (kNN). We employed the ADASYN strategy to improve the classifier performance in the imbalanced dataset. We performed k‐fold cross‐validation used as a resampling method (*k* = 10) on the training set and the hyperparameters were tuned by grid search. The validation set was used to adjust for the model parameters, whereas the test set was used to evaluate the performance of the system. The clinical value of this prediction model was evaluated by three model quality measurements, including discrimination, calibration, and clinical usefulness. First, model discrimination was quantified using precision‐recall curve analysis. Subsequently, we assessed the model's performance using calibration plots to assess how far the calibration and model predictions deviated from actual events. Then, the clinical utility was evaluated using DCA, which calculated the net benefits for various threshold probabilities. Furthermore, the confusion matrix metrics of AP, accuracy, sensitivity, specificity, and F1 score were assessed for the six models.

### 2.4 Model interpretation

It is well‐established that the application of machine learning techniques is limited by the difficulty of interpreting the results. The SHAP method proposed by Lundberg et al. is a game theoretic method to explain the output of any machine learning model and is reliable, fast, and computationally cheap.^15^ Importantly, the SHAP approach is used to sort the importance of each predictor based on its SHAP value. The output of the ML model is positively influenced by high SHAP values and vice versa.

### 2.5 Statistical analysis

Software including R (version 3.6.8), Python (version 3.7), and SEER*Stat (https://seer.cancer.gov/seerstat/) were used in this study. The used packages were shown in Table 2.

**TABLE 2:** Detailed information about the packages used in the development of machine learning models

Package name	Version	Description
Numpy	1.19.5	Numpy is the fundamental package for array computing with python
Pandas	1.0.4	Powerful data structures for data analysis, time series, and statistics
Matplotlib	3.3.2	Python plotting package
Sklearn	0.22.1	A set of python modules for machine learning and data mining
XGBoost	1.2.1	A set of python modules for machine learning and data mining
Imblean	0.0	Toolbox for imbalanced dataset in machine learning
PDPbox	0.2.1	Python partial dependence plot toolbox
SHAP	0.39.0	Toolbox for exploring how the input features contribute to the output of a complex machine learning model

## 3 RESULTS

### 3.1 Patient characteristics

This study enrolled a total of 2444 individuals with osteosarcoma, with 553 having distant metastases and 1891 not having metastasis. Table 3 shows the comprehensive demographic and clinical information.

**TABLE 3:** Demographic and clinicopathologic variables of the whole cohort grouped by metastasis status

Variables	All (*n* = 2444)	Distant metastasis (−) (*n* = 1891)	Distant metastasis (+) (*n* = 553)	*p*
Age				<0.001
0 –24	1567 (64.116)	1214 (64.199)	353 (63.834)	
25–59	662 (27.087)	543 (28.715)	119 (21.519)	
>60	215 (8.797)	134 (7.086)	81 (14.647)	
Size				<0.001
<50 mm	374 (15.303)	342 (18.086)	32 (5.787)	
51‐99 mm	1025 (41.939)	819 (43.310)	206 (37.251)	
>100 mm	1045 (42.758)	730 (38.604)	315 (56.962)	
Race				<0.001
White	1840 (75.286)	1429 (75.568)	411 (74.322)	
Black	394 (16.121)	301 (15.918)	93 (16.817)	
Other	210 (8.592)	161 (8.514)	49 (8.861)	
Gender				<0.001
Male	1336 (54.664)	1005 (53.146)	331 (59.855)	
Female	1108 (45.336)	886 (46.854)	222 (40.145)	
Tumor site				<0.001
Axial	285 (11.661)	175 (9.254)	110 (19.892)	
Extremity	1913 (78.273)	1503 (79.482)	410 (74.141)	
Other	246 (10.065)	213 (11.264)	33 (5.967)	
Grade				<0.001
Grade I	94 (3.846)	91 (4.812)	3 (0.542)	
Grade II	172 (7.038)	154 (8.144)	18 (3.255)	
Grade III	770 (31.506)	579 (30.619)	191 (34.539)	
Grade IV	1408 (57.610)	1067 (56.425)	341 (61.664)	
Histology				<0.001
9180	1596 (0.653)	1190 (0.629)	406 (0.734)	
9181	361 (0.148)	282 (0.282)	79 (0.143)	
9182	114 (0.470)	99 (0.520)	15 (0.270)	
9183	84 (0.340)	69 (0.360)	15 (0.270)	
9184	15 (0.600)	9 (0.500)	6 (0.110)	
9185	23 (0.900)	15 (0.800)	8 (0.140)	
9186	86 (0.35)	74 (0.390)	12 (0.220)	
9187	6 (0.200)	6 (0.300)	0	
9192	111 (0.450)	104 (0.550)	7 (0.130)	
9193	34 (0.140)	33 (0.170)	1 (0.200)	
9194	14 (0.600)	10 (0.500)	4 (0.700)	
Primary tumor surgery				<0.001
No	310 (12.684)	131 (6.928)	179 (32.369)	
Yes	2134 (87.316)	1760 (93.072)	374 (67.631)	
Radiotherapy				<0.001
No	2234 (91.408)	1767 (93.443)	467 (84.448)	
Yes	210 (8.592)	124 (6.557)	86 (15.552)	
Chemotherapy				<0.001
No	433 (17.717)	363 (19.196)	70 (12.658)	
Yes	2011 (82.283)	1528 (80.804)	483 (87.342)	
Lymphadenectomy				<0.001
No	2177 (89.075)	1673 (88.472)	504 (91.139)	
Yes	267 (10.925)	218 (11.528)	49 (8.861)	

### 3.2 Feature analysis

**FIGURE 1:**
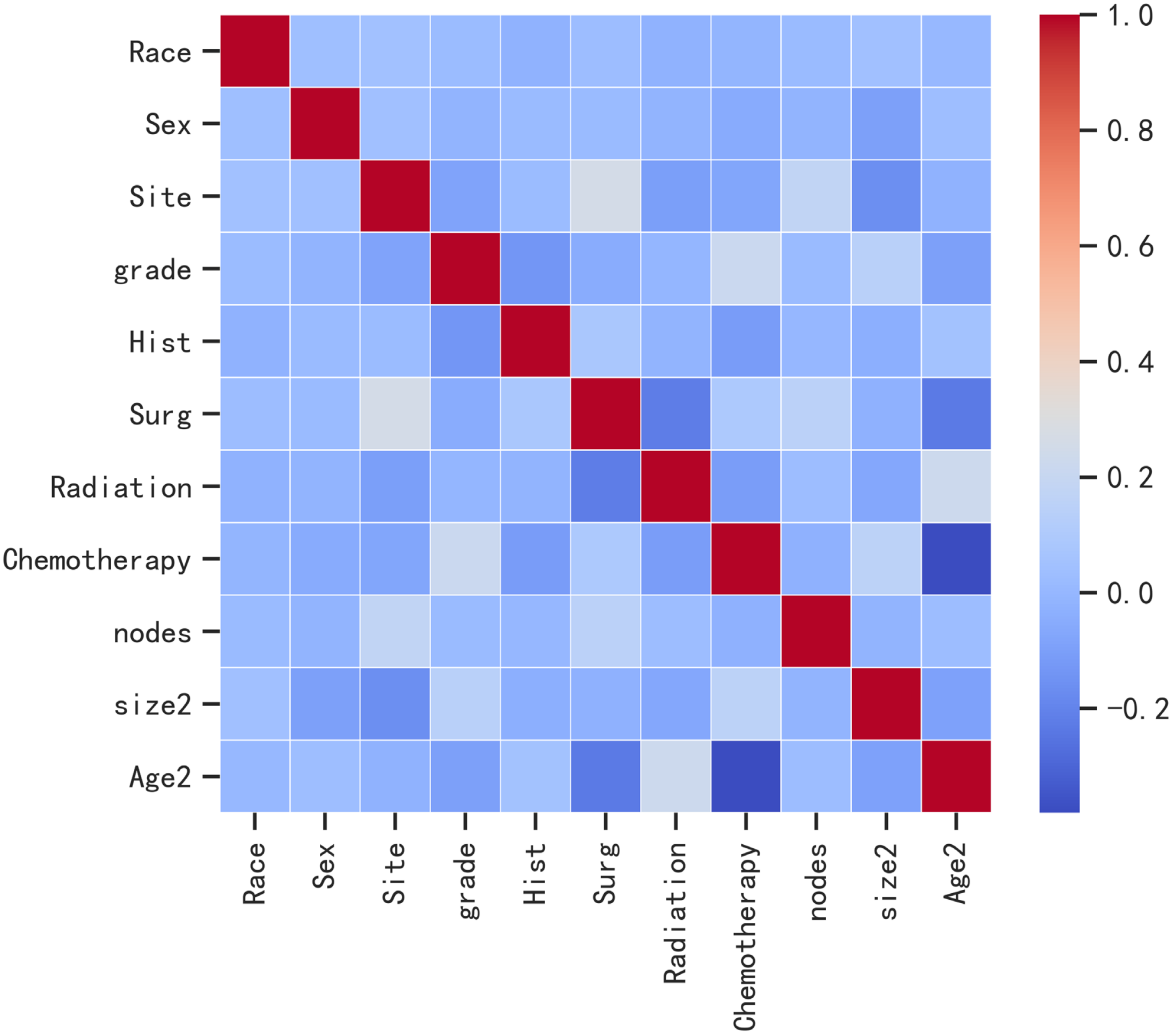
Correlation between factors.

Pearson correlation analysis was used to examine the relationship between each variable. The correlation heatmap (Figure 1) revealed a weak relationship between age and chemotherapy. Table 4 shows the results of machine learning classification algorithms applied on the balanced datasets generated with ADASYN techniques. Importantly, the ADASYN approach significantly enhanced the AP values of the classification models. The RF classifier achieved the highest precision values in the validation set for the 100% increase (0.688), the 150% increase (0.732) and the 200% increase (0.781) in validation set.

**TABLE 4:** Evaluation of the performance of classification models on imbalance dataset using ADASYN technique in validation set

Model	ADASYN	Precision	Accuracy (%)	Sensitivity (%)	Specificity (%)	F1‐score
XGBoost	100%	0.655 (0.635–0.676)	0.693 (0.676–0.710)	0.699 (0.636–0.761)	0.706 (0.630–0.781)	0.619 (0.592–0.647)
	150%	0.694 (0.677–0.711)	0.687 (0.677–0.697)	0.696 (0.659–0.733)	0.707 (0.670–0.745)	0.659 (0.640–0.678)
	200%	0.757 (0.740–0.775)	0.711 (0.701–0.721)	0.675 (0.653–0.696)	0.763 (0.738–0.788)	0.681 (0.665–0.697)
LR	100%	0.553 (0.511–0.595)	0.614 (0.598–0.630)	0.666 (0.588–0.743)	0.618 (0.541–0.695)	0.550 (0.511–0.590)
	150%	0.547 (0.514–0.579)	0.616 (0.604–0.628)	0.572 (0.452–0.692)	0.661 (0.584–0.737)	0.547 (0.481–0.613)
	200%	0.663 (0.653–0.673)	0.613 (0.599–0.628)	0.687 (0.613–0.761)	0.571 (0.514–0.628)	0.626 (0.588–0.663)
RF	100%	0.688 (0.666–0.711)	0.703 (0.692–0.714)	0.623 (0.572–0.673)	0.800 (0.755–0.845)	0.595 (0.569–0.621)
	150%	0.732 (0.717–0.746)	0.708 (0.697–0.720)	0.690 (0.678–0.701)	0.746 (0.735–0.758)	0.671 (0.660–0.681)
	200%	0.781 (0.766–0.795)	0.718 (0.710–0.726)	0.687 (0.659–0.715)	0.781 (0.751–0.812)	0.688 (0.667–0.708)
GaussianNB	100%	0.575 (0.556–0.594)	0.609 (0.592–0.627)	0.453 (0.374–0.531)	0.819 (0.743–0.895)	0.458 (0.411–0.504)
	150%	0.626 (0.604–0.647)	0.631 (0.616–0.647)	0.635 (0.563–0.707)	0.641 (0.565–0.718)	0.614 (0.565–0.662)
	200%	0.661 (0.640–0.682)	0.619 (0.609–0.628)	0.553 (0.450–0.656)	0.710 (0.609–0.812)	0.576 (0.505–0.646)
kNN	100%	0.636 (0.620–0.651)	0.692 (0.681–0.703)	0.705 (0.629–0.781)	0.680 (0.606–0.753)	0.623 (0.588–0.658)
	150%	0.684 (0.670–0.698)	0.698 (0.687–0.709)	0.659 (0.591–0.727)	0.737 (0.672–0.802)	0.665 (0.629–0.701)
	200%	0.717 (0.707–0.727)	0.697 (0.693–0.702)	0.740 (0.693–0.787)	0.668 (0.623–0.714)	0.719 (0.695–0.743)
SVM	100%	0.631 (0.611–0.652)	0.646 (0.630–0.662)	0.664 (0.606–0.722)	0.655 (0.595–0.716)	0.575 (0.544–0.606)
	150%	0.644 (0.629–0.659)	0.643 (0.629–0.658)	0.652 (0.574–0.730)	0.658 (0.577–0.739)	0.608 (0.569–0.647)
	200%	0.678 (0.658–0.699)	0.637 (0.631–0.643)	0.661 (0.596–0.725)	0.624 (0.560–0.689)	0.628 (0.599–0.657)

### 3.3 Model development and evaluation

**FIGURE 2:**
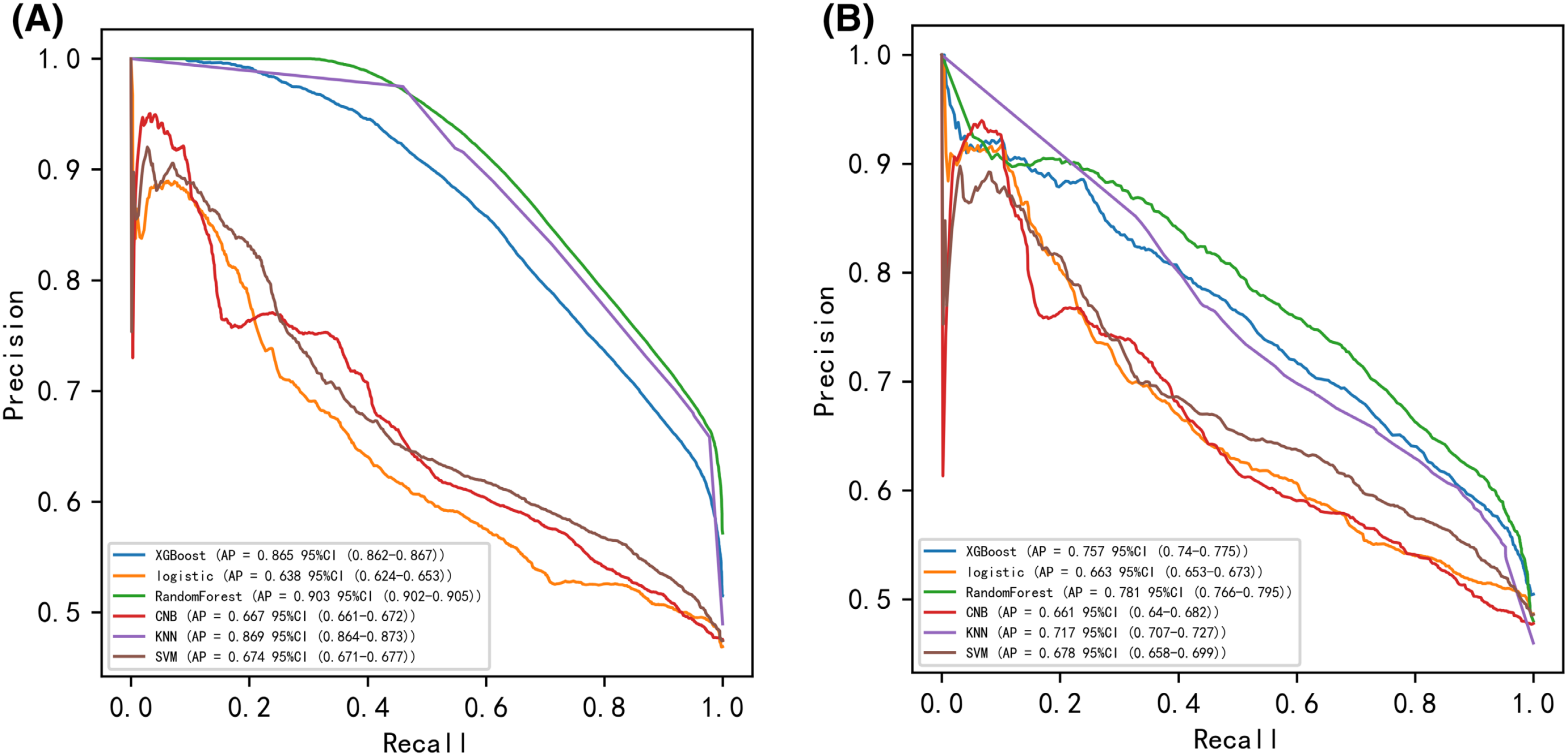
Evaluation of the prediction model for distant metastasis in Osteosarcoma, the average precision recall curves, indicating the trade off between precision and recall.

**FIGURE 3:**
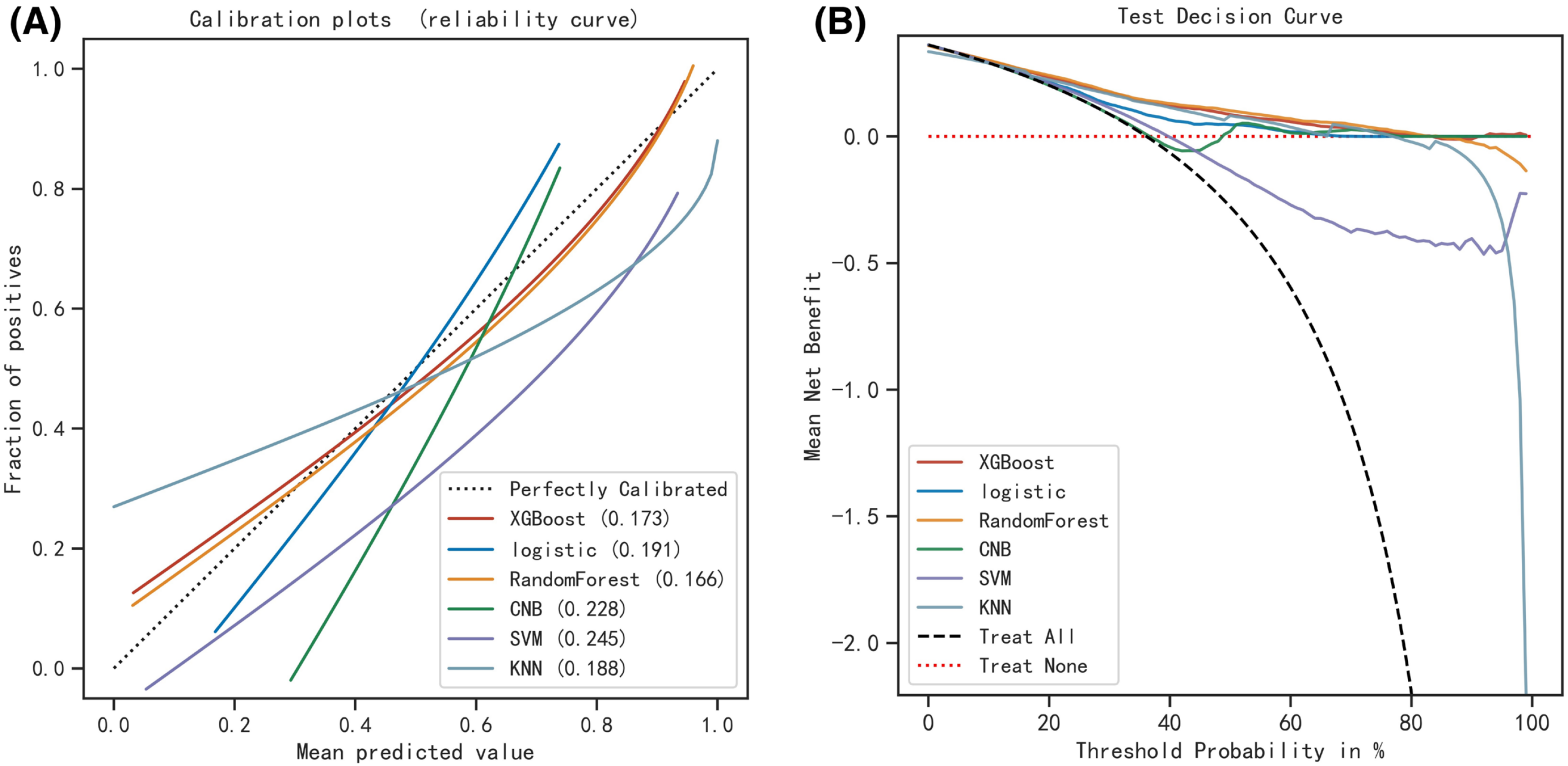
Examples of calibration plots for predicting LNI with various models: XGB, LR,RF, GaussianNB, SVM, and kNN. (A) the test set. (A) The 45° straight line on each graph represents the perfect match between the observed (y‐axis) and predicted (x‐axis) survival probabilities. A closer distance between two curves indicates greater accuracy Decision curve analysis graph showing the net benefit against threshold probabilities based on decisions from model outputs. (B) Three curves were obtained based on predictions of the four different models, and the two curves obtained were based on two kinds of extreme decisions. The curves referred to as“All” represent the prediction that all the patients would progress to DM, and the curves referred to as “None” represent the prediction that all the patients were DM.

**FIGURE 4:**
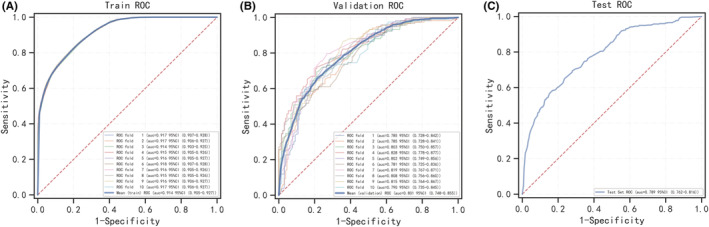
ROC curves of RF for the training, validation and test group.

Six machine learning algorithms were employed to build a prediction model in this study. The training set was used to create and train machine learning models. After adjusting for parameters and comparing algorithms, the average precision values of six machine learning models were greater than 0.64, indicating that predictive models have good predictive ability. (Figure 2). As described in Figure 2, the RF algorithm yielded the best prediction performance in precision‐recall curves between the training and validation set. The precision‐recall curves, calibration plots, and DCAs for the validation set were generated to evaluate the prediction model. The calibration plots of the validation set indicated that the predictive probabilities against observed the risk of distant metastasis (Figure 3A). DCA of the six models was subsequently conducted, showing that all models achieved net clinical benefit against a treat all‐or‐none plan, except kNN (Figure 3B). Regarding calibration plots and DCAs, the RF model also performed best. Table 4 shows the confusion matrix evaluation measures as well as the average precision of all prediction models. All prediction models' k‐fold cross‐validation accuracies (k = 10) are listed in Table 5. Our results showed that the RF model yielded the highest k‐fold cross‐validation accuracy. Among these, the predictive model using the RF algorithm yielded the best predictive performance. (Figure 4).

**TABLE 5:** The k‐fold cross‐validation accuracies (k = 10) of all six prediction models

Model	XGB	RF	Gaussian NB	kNN	SVM	LR
k‐fold accuracy	0.711 (0.701–0.721)	0.718 (0.710–0.726)	0.619 (0.609–0.628)	0.697 (0.693–0.702)	0.637 (0.631–0.643)	0.613 (0.599–0.628)

### 3.4 Model interpretation

**FIGURE 5:**
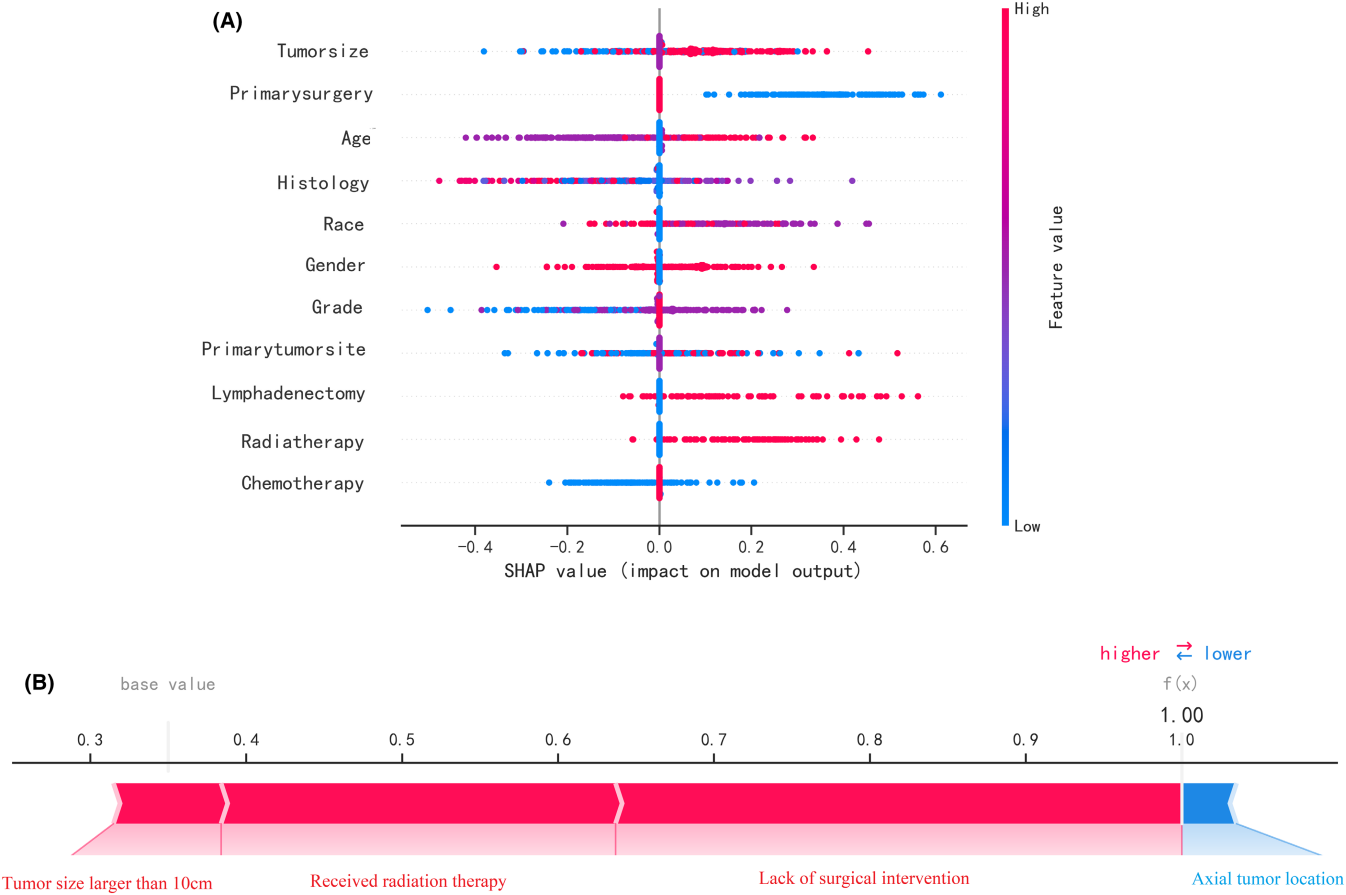
Summary plots for SHAP values. For each feature, one point corresponds to a single patient. (A) A point's position along the x axis (i.e., the actual SHAP value) represents the impact that feature had on the model's output for that specific patient. Mathematically, this corresponds to the (logarithm of the) mortality risk relative across patients (i.e., a patient with a higher SHAP value has a higher mortality risk relative to a patient with a lower SHAP value). Features are arranged along the y axis based on their importance, which is given by the mean of their absolute Shapley values. The higher the feature is positioned in the plot, the more important it is for the model. Explained risk for individual. (B) The contributing variables are arranged in the horizontal line, sorted by the absolute value of their impact. The output value is the predicted risk of lymph node metastasis. The base value means the expected value of model C, over the training dataset.

Figure 5A depicts the predictive model's SHAP summary plot, which consists of 11 features sorted by their impact on metastatic status. The higher the SHAP value of a feature, the higher the risk of distant metastasis. The red color denotes a high feature value, purple denotes a feature value close to the overall average, and blue denotes a low feature value. Figure 5B provides an example for predicting the risk of metastasis in an osteosarcoma patient. In this case, the RF model predicted a distant metastasis risk of 1.00 (base value: 0.35). The probability of distant metastasis was increased by tumor size (more than 10 cm), radiation therapy, and lack of surgical intervention. The axial placement of the tumor decreased the estimated probability of metastasis. The axial tumor location reduced the predicted risk of metastasis.

## 4 DISCUSSION

Patients with osteosarcoma who develop distant metastasis have a significantly lower overall survival rate, with a 5‐year survival rate of only 20%.^16^ It has been established that patients with distant metastasis derive little benefit from surgery, chemotherapy, or novel immunotherapy, accounting for the poor prognosis of advanced osteosarcoma patients.^17^ As a result, it is critical to identify risk factors associated with distant metastasis in osteosarcoma patients to facilitate early detection, prevention, and prognosis assessment.

An ML‐based model was trained and validated in this study to predict the risk of distant metastasis. This ML model was based on clinical data, including age, gender, race, tumor size, primary tumor site, grade, histology, surgery, radiotherapy, chemotherapy, lymphadenectomy, and stage. The ADASYN technique was then used to deal with imbalanced data, yielding a more balanced dataset. As a result, the RF model outperformed all other models in training and validation sets. To improve the interpretability of our machine learning model, we reported the SHAP values and a list of highly influential features.

We see at least two approaches to clinical practice implementation. To begin, individual doctors and patients could use the model to assess risk and make tailored decisions about osteosarcoma screening in the clinic. Second, health systems could use the model to identify high‐risk patients for additional outreach at the population level.

In the present study, SHAP analysis demonstrated that the top three informative features in the model were tumor size, primary surgery, and age. The present study consistently showed that tumor size is an important risk factor. Moreover, it has been reported that a large tumor at the time of diagnosis is associated with a greater number of lung metastases.^18^ Interestingly, tumor size has been shown to be an independent predictor of overall survival and distant metastases in patients with osteosarcoma using plain radiographs or magnetic resonance imaging.^9^ In this regard, tumors treated without surgery, with higher pathological grades and larger size, contributed to an increased risk of cancer metastasis. Surgery, which involves removing the tumor or amputating, remains the only effective treatment for osteosarcoma and is not indicated in patients with distant metastasis.^19^ Although the efficacy of surgery is variable due to various factors, radical resection of the primary tumor inhibits tumor progression, including metastases, to a certain extent.^20^, ^21^ Our findings indicate that the absence of surgery exerted a significant negative effect on distant metastasis, which corroborates the above findings. Although age as risk factor for distant metastases are less intuitive than primary surgery and tumor size has been previously found to be risk factors for advanced disease in in studies of other malignancies.^22^, ^23^ It had been found in a large study that includes over 27,000 patients that older age was an independent significant risk factor for distant metastases.^24^ Similarly, a recent study had also discovered that older age at diagnosis was at a higher risk of developing pleural metastases.^25^ In concordance with these studies, our results find that a high risk of distant metastasis among patients older than 60 years. In this study, we divided patients into separated into different age groups (<24 years old, 24–59 years old, and >60 years old) according to recent publication.^26^ The reasons for this may be related to health access, among other socioeconomic features.

Previous efforts to improve osteosarcoma prognostic prediction were based on parametric regression models, widely used in clinical studies due to their ease of use and interpretability. Regression analysis, on the other hand, can only be carried out in its entirety.^27^ Importantly, regression modeling assumes linear and homogeneous relationships among input variables. Nonetheless, it should be borne in mind that complex interactions exist between predictors.^28^ Decision tree‐based methods are a subset of machine learning algorithms that uncover complex non‐linear relationships between covariates.^29^ The final class of an instance in a random forest is determined by outputting the class that is the mode of individual tree outputs, resulting in robust and accurate classification as well as the ability to handle a large number of input variables.^30^ Random forests are relatively resistant to overfitting and are capable of handling datasets with highly asymmetric class distributions.^31^ In the training and validation sets, our AI model based on RF had a high prediction performance for the risk of distant metastasis in osteosarcoma patients (AP = 0.903 and 0.781, respectively). The retrospective nature of this study, however, limited it, and selection bias was inevitable. Moreover, we could not identify socioeconomic factors linked to patient survival and the occurrence of pathological fractures in osteosarcoma. Finally, “no” and “unknown” were combined into one group in the SEER data for chemotherapy and radiation. Chemotherapy and radiation were significantly underreported, which we could not overlook.

In conclusion, we employed the random forest method to develop an artificial intelligence model for predicting the probability of distant metastasis in patients with osteosarcoma. According to precision‐recall analysis, our prediction model yielded accurate results. DCA demonstrated that the model provided a net benefit. Our findings substantiate that this prediction model has huge prospects for application during clinical practice to assist physicians in making more informed treatment decisions for osteosarcoma patients.

## AUTHOR CONTRIBUTIONS

Conceptualization, BL.B. and Q.Y.; Methodology, ZY.W. and SJ.W.; Software, SJ.W.; Investigation, BL.B.;Resources, BL.B. and Q.Y.; Data curation, Q.Y.; Formal analysis, ZY.W., SJ.W. and BL.B.; Validation, BL.B. and Q.Y.; Writing‐original draft preparation, ZY.W., SJ.W., and Q.Y.; Writing—review and editing, BL.B. and Q.Y.; Visualization, BL.B.; Supervision, Q.Y.

## FUNDING INFORMATION

This research did not receive any specific grant from funding agencies in the public, commercial, or not‐ for‐ profit sectors.

## CONFLICT OF INTEREST

We have read and understood Cancer Medicine's policy on disclosing conflicts of interest and declare that we have none.

## ETHICAL APPROVAL STATEMENT

We received permission to access the research data file in the SEER program from the National Cancer Institute, US. Approval was waived by the local ethics committee, as SEER data are publicly available and de‐identified.

## Data Availability

The datasets generated during and/or analyzed during the current study are available from the corresponding author on reasonable request.
